# A Novel Creatinine-Based Equation to Estimate Glomerular Filtration Rate in Chinese Population With Chronic Kidney Disease: Implications for DOACs Dosing in Atrial Fibrillation Patients

**DOI:** 10.3389/fphar.2021.615953

**Published:** 2021-02-19

**Authors:** Ling-Yun Zhou, Wen-Jun Yin, Jun Zhao, Bi-Kui Zhang, Can Hu, Kun Liu, Jiang-Lin Wang, Ge Zhou, Lin-Hua Chen, Shan-Ru Zuo, Yue-Liang Xie, Xiao-Cong Zuo

**Affiliations:** ^1^ Department of Pharmacy, The Third Xiangya Hospital, Central South University, Changsha, China; ^2^ Department of Clinical Pharmacy, First Affiliated Hospital of Xinjiang Medical University, Urumqi, China; ^3^ Department of Pharmacy, Second Xiangya Hospital, Central South University, Changsha, China; ^4^ Center of Clinical Pharmacology, The Third Xiangya Hospital, Central South University, Changsha, China

**Keywords:** chronic kidney disease, atrial fibrillation, anticoagulants, dose adjustment, Xiangya-s equation, clinical outcomes

## Abstract

**Background:** Over/under-estimating renal function may increase inappropriate dosing strategy associated adverse outcomes; however, previously reported equations to estimate renal function have limited accuracy in chronic kidney disease (CKD) patients. Consequently, we intended to develop a novel equation to precisely estimate renal function and subsequently guide clinical treatment for CKD patients.

**Methods:** A novel approach, Xiangya-s equation, to estimate renal function for CKD patients was derived by linear regression analysis and validated in 1885 patients with measured glomerular filtration rate (mGFR) < 60 ml/min/1.73 m^2^ by renal dynamic imaging at three representative hospitals in China, with the performance evaluated by accuracy, bias and precision. In the meanwhile, 2,165 atrial fibrillation (AF) patients who initiated direct oral anticoagulants (DOACs) between December 2015 and December 2018 were identified and renal function was assessed by estimated creatinine clearance (eCrCl). Events per 100 patient-years was calculated. Cox proportional hazards regression was applied to compare the incidence of outcomes of each group.

**Results:** Xiangya-s equation demonstrated higher accuracy, lower bias and improved precision when compared with 12 creatinine-based and 2 CysC-based reported equations to estimate GFR in multi-ethnic Chinese CKD patients. When we applied Xiangya-s equation to patients with AF and CKD prescribed DOACs, wide variability was discovered in eCrCl calculated by the Cockcroft-Gault (CG), Modification of Diet in Renal Disease Study (MDRD), Chronic Kidney Disease Epidemiology Collaboration (CKD-EPI), Xiangya equation which we had developed for generally patients and Xiangya-s equations, which persisted after grouping by different renal function stages. Equation choice affected drug-dosing adjustments, with the formulas agreeing for only 1.19%, 5.52%, 33.22%, 26.32%, and 36.61% of potentially impacted patients for eCrCl cutoffs of <15, <30, 15–49, 30–49, ≥50 ml/min, respectively. Relative to CG equation, accordance in DOACs dosage was 81.08%, 88.54%, 62.25%, and 47.68% for MDRD, CKD-EPI, Xiangya and Xiangya-s equations for patients with CrCl < 50 ml/min (eCrCl cutoffs of <30, 30–49, ≥50 ml/min), respectively. Reclassification of renal function stages by Xiangya-s equation was significantly associated with stroke or systemic embolism, non-major clinically relevant bleeding and any bleeding events.

**Conclusion:** Xiangya-s equation provides more accurate GFR estimates in Chinese CKD patients who need consecutive monitoring of renal function, which may assist clinicians in choosing appropriate drug dosages.

## Introduction

Chronic kidney disease (CKD) has been recognized as one of the independent risk factors of cardiovascular disease, end-stage renal disease, and mortality, even in its early stages (Eckardt et al., 2013). In China, the overall prevalence of CKD was reported to be 10.8% (Zhang et al., 2012). Glomerular filtration rate (GFR) is accepted as the preferred index of renal function and to define CKD (a reduced GFR <60 ml/min/1.73 m^2^ for more than 3 months) (Foundation, 2002). Many methods have been developed to directly measure GFR, including exogenous and endogenous filtration markers clearance by analytical techniques (Foundation, 2002). Directly measured GFR (mGFR) obtained using 99m-technetium-diethylenetriaminepentaacetic acid (99mTc-DTPA) renal dynamic imaging, proposed by the Nephrology Committee of Society of Nuclear Medicine (Blaufox et al., 1996), was reported to be close to the inulin clearance rate of 0.99 over a wide range of GFR, confirming similar renal handling, and has been identified as the reference in most regions of China (Pei et al., 2013). However, its high cost and tedious operation make it not easily available in most clinical settings in the developing countries.

As an alternative, estimated creatinine clearance (eCrCl) (Steffel et al., 2018) or estimated glomerular filtration rate (eGFR) (Levey et al., 2009) was calculated to indirectly estimate kidney function, both of which have been used to assist in making dosing decision in clinical practice for decades. However, the optimal method to assess an individual’s kidney function remains controversial (Bukabau et al., 2019; Selistre et al., 2019). The National Kidney Education Program recommended that eCrCl calculated by Cockcroft–Gault (CG) equation and eGFR calculated by Modification of Diet in Renal Disease Study (MDRD) equation could be used interchangeably for drug dosing, while the Kidney Disease: Improving Global Outcomes suggested that Chronic Kidney Disease Epidemiology Collaboration (CKD–EPI) equation may be a proper choice for staging CKD (Pottel et al., 2019). However, many studies have (Li et al., 2019; Yue et al., 2020) found that none of the published methods can precisely estimate the renal function in Asian patients with CKD. Even our previously developed equation, Xiangya equation which have been proved to provide more accurate GFR estimates in Chinese adults and can replace existing eGFR equations for use in the Chinese population, could not satisfying enough for patients with CKD in the internal and external validation sets (Li et al., 2019; Yue et al., 2020).

Roughly 50% of all drugs or their metabolites are excreted by the kidneys (Hartmann et al., 2010), and failure to accurately assess individual’s renal function may lead to 23% (Frölich et al., 2011) inappropriate drug dosage adjustment in CKD patients and significantly increase the risk of mortality by 40%, leading to increased health care utilization costs (Breton et al., 2011). Until recently, many studies have performed to compare the different drug dosing regimens based on different equations to estimate renal function (Manzano-Fernández et al., 2015; Lee et al., 2019a). For example, patients with atrial fibrillation (AF) who need oral anticoagulants, including vitamin K antagonists (VKAs) and direct oral anticoagulants (DOACs), are considered to be at high risk of renal function related adverse events (Roldán et al., 2013a; Roldán et al., 2013b; Esteve-Pastor et al., 2018). The presence of impaired renal function was closely associated with thrombotic/vascular events, bleeding, and mortality in AF patients with anticoagulant treatment (Roldán et al., 2013a). Patients with moderate CKD were at the risk of future major hemorrhagic events in AF patients. Different definition of renal function could markedly improve the predictive ability of hemorrhagic risk stratification of HAS-BLED score (Suzuki et al., 2014). Although adding CKD to the CHADS_2_ and CHA_2_DS_2_-VASc stroke risk scores was found to not independently add predictive information (Roldán et al., 2013b), the presence of severe CKD was an independent factor for the clinical adverse outcomes in AF patients and worsening CrCl is an excellent independent predictor of ischemic stroke/systemic embolism and bleeding (Hindricks et al., 2020). Additionally, In the FANTASIIA registry (Esteve-Pastor et al., 2018), a prospective and real-world AF registry, approximately 67% of patients with severe CKD had poor anticoagulation control while taking VKAs. Furthermore, all of the direct oral anticoagulants (DOACs), the first-line therapy to prevent atrial fibrillation (AF)-associated ischemic events (Chiang et al., 2014; Kirchhof et al., 2016; January et al., 2019; Hindricks et al., 2020), have some dependence on renal elimination (Steinberg and Washam, 2017), contributing to the requirement of dose adjustment in CKD patients to avoid drug accumulation if overdosed and thromboembolic events if underdosed, especially for those with a greater dependence on renal clearance (dabigatran, rivaroxaban) (Steffel et al., 2018). Renal-based dosing makes it an additional challenge in clinical practice, as accurate estimation of renal function is not easily achievable (Levey et al., 2009; Manzano-Fernández et al., 2015; Lee et al., 2019a). The European/American/Asian guidelines (Chiang et al., 2016, Chiang et al., 2017; January et al., 2019; Hindricks et al., 2020) regarding the management of AF patients all recommended that timing and accurate assessment of renal function is important for the appropriate treatment of AF patients, especially for the dosing adjustment of DOACs. Although the landmark DOACs clinical trials only used eCrCl calculated with the CG formula, not eGFR, to assess renal function, the eGFR estimated by CKD-EPI (Steffel et al., 2018) or MDRD (Levey et al., 2009) is more frequently used to make dosing decision in clinical practice as recommended by the guidelines regarding the management of CKD patients. What’s more, some studies have revealed the discordance in DOACs dosages using different equations, especially among patients with renal impairment (Manzano-Fernández et al., 2015). Thus, the optimal method to assess an individual renal function to guide DOACs dosage adjustment remains controversial.

Consequently, the aim of the present study was to develop a novel approach based on a multi-center, multi-ethnic Chinese large CKD population, so as to accurately estimate GFR for patients with CKD, and then to evaluate the clinical implications of the novel approach in AF patients treated with AF.

## Materials and Methods

### Study Population

**FIGURE 1:**
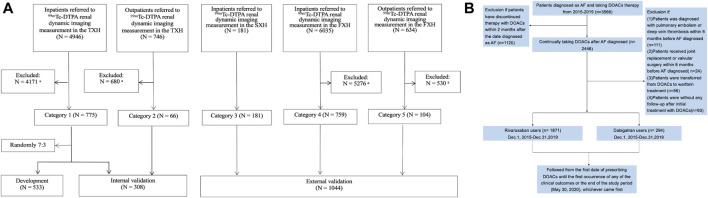
Flow charts of present study showing: **(A)** development and validation of the new equation pooled creatinine database; **(B)** enrollment of AF Patients in our study. **(A)**: Data from TXH and FXH were collected by using the database systems of both hospitals according to the same inclusion and exclusion criteria for screening populations, and the part from SXH was strictly extracted following the inclusion criteria in our research. ^99m^Tc-DTPA, ^99m^Tc-diethylene triamine pentaacetic acid; GFR, glomerular filtration rate; mGFR, measured GFR; TXH, the Third Xiangya Hospital of Central South University; SXH, the Second Xiangya Hospital of Central South University; FXH, the First Affiliated Hospital of Xinjiang Medical University. ^a^Further detail of patients excluded according to the exclusion criteria are shown in Supplementary Table S1. **(B)**: A total of 2,165 patients with nonvalvular AF was included. AF, atrial fibrillation; DOACs, direct oral anticoagulants.

Patients with mGFR<60 ml/min/1.73 m^2^ extracted from a cohort of 8,561 patients referred to 99mTc-DTPA renal dynamic imaging in 3 representative Class-III/Grade-A hospitals of China as described previously (Li et al., 2019), consisting of the Third Xiangya Hospital of Central South University (TXH) from July 2010 to May 2017 and the Second Xiangya Hospital of Central South University (SXH) from August 2011 to December 2017 in Hunan (the middle of China), and the First Affiliated Hospital of Xinjiang Medical University (FXH) from August 2011 to June 2017 in Xinjiang (the northwest of China, with multiple ethnicities) were included (Figure 1A). The exclusion criteria for screening patients were as follows: 1) being aged <18 years; 2) receiving dialysis treatment at the time of the study; 3) having incomplete data for mGFR; 4) having missing baseline values for serum creatinine level; 5) taking medicine that significantly affects creatinine level, within 10 days prior to GFR measurement; and 6) having a creatinine level >700 mmol/L. The detailed information was shown in Supplementary Table S1. The novel equation was developed and internal-validated in TXH, and external-validated in SXH and FXH.

In the meanwhile, AF patients with CHA_2_DS_2_-VASc≥2, with no other indications for DOACs treatment, who initiated DOACs between December 1, 2015 and December 31, 2019 were identified in TXH center, to validate the performance of the novel equation in real-world clinical practice (Figure 1B). As guideline recommended, DOACs should be avoided in patients with valvular heart disease, such as rheumatic mitral stenosis, a mechanical or bioprosthetic heart valve or mitral valve repair. Thus, we excluded patients with these indications. Also, patients undergoing hip or knee replacement surgery and having a diagnosis of deep vein thrombosis or pulmonary embolism were ineligible in present study.

### Laboratory Measurements and Clinical Definitions

Information on demographics, diagnosis, and medications of each admission were obtained from the hospital electronic medical records of each center. Core elements of the data warehouse are completely de-identified so that all queries and analytics can be carried out without exposing private health data and informed consent was waived for this study. Institutional Ethical Committee approval was obtained (No. 2017-S292). GFR was measured using 99mTc-DTPA renal dynamic imaging by Ifinia Hawkeye four SPECT (GE Healthcare, United States) (Li et al., 2019).

When applying the novel approach to AF patients treated with DOACs, the most recent serum creatinine (SCr) levels within 1 week before treatment initiation were abstracted. As guideline and product monographs recommended, eCrCl was used to assess individual renal function and the CG equation was regarded as the “gold standard” (Steffel et al., 2018). Also, we calculated eGFR using CKD-EPI (Steffel et al., 2018), MDRD (Levey et al., 2009), Xiangya equation (Li et al., 2019) and the novel formula. The reasons why we chose the other four equations were as follows: 1) Xiangya equation was our previously developed equation for Chinese general patients, which has been proved to can provide more accurate GFR estimates in Chinese adults and replace existing eGFR equations for use in the Chinese population (Li et al., 2019). 2) CG equation was the only equation which the landmark DOACs clinical trials used to calculate eCrCl to guide dosage adjustments of DOACs (Connolly et al., 2009; Granger et al., 2011; Patel et al., 2011; Giugliano et al., 2013). Also, the guidelines regarding the use of DOACs in patients with AF recommended CG equation to be the choice in clinical practice (Chiang et al., 2016; Steffel et al., 2018; January et al., 2019; Hindricks et al., 2020). Thus, in present study, we used the CG equation as the reference. 3) MDRD and CKD-EPI equations were two of the most frequently used equations to estimate GFR in the Chinese real-world clinical practice, as endorsed by the guidelines regarding the management of CKD patients (Foundation, 2002; Stevens et al., 2013).

Consequently, eGFR was adjusted by individual’s body surface area. Patients were assigned to different renal function stages based on each equation as the guideline recommended (Steffel et al., 2018). As no patients were treated with apixaban or edoxaban in present cohort, patients were considered to have a renal indication for dose reduction if they were prescribed dabigatran and had a CrCl<30 ml/min, rivaroxaban and a CrCl <50 ml/min (Steffel et al., 2018). Meanwhile, co-treatment with medications, which may have drug interactions with P-glycoprotein and cytochrome P450 3A4 inhibitors based on guideline, was not used as the criteria for dose reduction because they are generally considered relative indications, and the effects on DOACs plasma levels vary substantially among patients and drugs (Steffel et al., 2018). Patients for whom the selected drugs were contraindicated were classified as overdosed (Supplementary Table S2).

The index date was defined as the first date of prescribing DOACs after diagnosed as AF. The follow-up period was defined from the index date until the first occurrence of any of the clinical outcomes or the end date of the study period (May 30, 2020), whichever came first. The clinical outcomes in present study were in consistent with what in the pivotal clinical trials of DOACs (Connolly et al., 2009; Granger et al., 2011; Patel et al., 2011; Giugliano et al., 2013; Steffel et al., 2018). The primary effectiveness outcome was ischemic stroke or systemic embolism (S/SE). The primary safety outcome was major bleeding as defined by the International Society on Thrombosis and Hemostasis (Steffel et al., 2018). The other outcomes are non-major clinically relevant bleeding, and any bleeding (Steffel et al., 2018). Clinical outcomes were independently collected by three researchers (Wen-Jun Yin, Jun Zhao, Bi-Kui Zhang). All discrepancies and uncertainties were resolved by consulting a fourth author (Xiao-Cong Zuo).

### Statistical Analysis

Baseline characteristics were presented as mean (SD) or median (interquartile range) for continuous variables, as appropriate, and percentages for categorical variables.

The detailed step for developing and validating the novel approach was similar with our previous study (Li et al., 2019). Briefly, for developing the new equation, we: 1) divided patients in the TXH center into 70% of the development sample and 30% of the internal validation dataset; 2) prespecified a process for developing equations that included natural logarithmic transformation, univariable analysis, and multivariable analysis: ①Firstly, mGFR was transformed to natural logarithms, and then a univariable analysis was performed to screen in the mGFR-related variables. Candidate variables included age, sex, weight, height, and laboratory test indicators. ②Secondly, original data, logarithmically or restricted cubic spline transformed continuous variables were used to reflect the multiplicative relationship and to stabilize variance across the range of GFR. ③A least-squares linear regression was performed to relate mGFR to the independent variables; 3) conducted the multivariable analysis modeling using the above filtered variables. A backward method was used to filter variables, and a set of candidate equations was developed; 4) selected the final equations among the candidate equations by comparing the *R* ^2^, adjusted *R* ^2^, Akaike’s information criterion (AIC) and the accuracy (mainly, P_30_) of these candidate models and the new equation, together with the ease of clinical use. Subsequently, internal- and external-validation was performed.

Then, we searched PubMed to find out eGFR equations which derived from Asian population using filtration markers as reference. The abstracts were evaluated by two independent reviewers, and if one or two researchers considered the publication to be potentially relevant, the full-text article would be reviewed. Additionally, clinical commonly used equations were supplemented. Bias, precision, and accuracy were used to assess the equation performance (Li et al., 2019), as proposed in K/DOQI (Foundation, 2002). Bias, a measure of systemic error, was defined by the mean difference between eGFR and mGFR (Levey et al., 2014). A positive value of bias indicates that the equation overestimates GFR, and a negative value indicates underestimation (Kilbride et al., 2013). Precision was defined as the inter-quartile range (IQR) of the difference between eGFR and mGFR to reflect the random variation of eGFR around the mGFR (Levey et al., 2014). Accuracy, expressing the proximity of the estimation compared with the reference, was calculated using two methods: root mean square error (RMSE) and the percentage of individuals with eGFR not deviating more than 30% from the mGFR (P_30_) (Itoh, 2003; Kilbride et al., 2013). Specifically, P_30_ encompassed both precision and bias, could measure vital errors in clinical practice with good consistency and stability, thus became the key indicator we focused on (Kilbride et al., 2013). According to the K/DOQI Guidelines, P_30_ ≥ 75% is considered sufficient to make good clinical decisions (Foundation, 2002).

When applying the novel approach in AF patients, percentage of patients with overdosed or underdosed DOACs were investigated. For the limited patients treated with underdosed DOACs, dosing patterns were transformed into a categorical variable with two levels (appropriately dosed, inappropriately dosed). What’s more, for the small number of patients treated with different DOACs, we pooled them together to increase power. Agreement between selected formulas and CG equation was inspected visually using Bland-Altman plots. The Venn diagrams were used to compare how many patients would meet the guidelines recommended eCrCl drug-dose-reduction cutoffs for DOACs to assess the impact of different equations on drug-dosing adjustments. Patients were assigned to different renal function stages as the guideline recommended (Steffel et al., 2018). Confusion matrices were used to investigate the extent of agreement in renal function staging by different eCrCl estimates, assessed with Cohen’s kappa. Furthermore, we used cox hazard regression model to investigate the association between different renal function stages estimated by selected equations and clinical outcomes.

## Results

### Characteristics of Participants

After screening the inclusive and exclusive criterions, 841 patients with mGFR < 60 ml/min/1.73 m^2^ in the TXH were enrolled with the mean age 58.23 ± 12.06 years for inpatients and 53.20 ± 11.07 years for outpatients. A random sample of 533 inpatients was included in the development set, and the remaining participants were included the internal validation set. Patients from the SXH (*n* = 285) and FXH (*n* = 759) were used for external validation. Characteristics of these individuals were summarized in Supplementary Tables S3 and S4.

In the AF cohort, rivaroxaban and dabigatran were taken by 1871 and 294 patients, respectively. Patients were followed up for a median of 18.5 months. Further details of these patients were available in Supplementary Table S5.

### Development, Internal-Validation, and External-Validation of Xiangya-s Equation

Similar with our previous study (Li et al., 2019), to evaluate the performance of the model, we focused on R^2^, adjusted R^2^, Akaike’s information criterion (AIC), and especially, clinical applicability. In general, the model with the smallest AIC value is preferred (Shipley, 2013). Eventually, the simplest model with 3 variables, including SCr concentrations, gender, and age, had an adjusted *R* ^2^ similar to that of the original model (0.6176 vs. 0.6278), suggesting that they have similar prediction accuracy, with the smallest AIC of 130.14. In additional, the model with 3 variables has greater clinical applicability than other models. Therefore, this model was selected as the best-performing equation. Table1 shows the Xiangya-s equation in form that could be implemented in clinical laboratories. Because the equation development is based on data from the TXH, we called it as the “supplementary Xiangya (Xiangya-s) equation”. In TXH center (Table 2), P_30_ of the inpatients was 79.61%, 78.99%, and 81.82% in the entire cohort, development sample, and internal validation dataset, respectively, which was considered to be sufficient for good clinical decision-making according to the 2002 K/DOQI benchmark (Foundation, 2002).

**TABLE 1:** The novel equation for estimating GFR in Chinese population with CKD^a^.

Name	Applicable population	Sex	Equation
Xiangya-s	Chinese	Male	eGFR = 627.2781 × SCr ^−0.38089^ × Age ^−0.18724^
		Female	eGFR = 627.2781 × SCr ^−0.38089^ × Age ^−0.18724^ × 0.9286438

Abbreviations: eGFR, estimated glomerular filtration rate; SCr, Serum creatinine; CrCl, creatinine clearance.

^a^ Expressed for specified mGFR level and sex. Serum creatinine was measured in mmol/l. To convert SCr from mmol/l to mg/dl, divide by 88.4.

**TABLE 2:** Performance of the Xiangya-s equation in patients with mGFR<60 ml/min/1.73 m^2^ of the development and internal validation cohort.

Characteristic and performance of Xiangya-s equation	Entire cohort		Development sample set	Internal validation sample set	
	Inpatients (*n* = 775)	Outpatients (*n* = 66)	Inpatients (*n* = 533)	Inpatients (*n* = 242)	Outpatients (*n* = 66)
Mean mGFR (SD), mL/min/1.73 m^2^	43.59 (12.08)	42.31 (13.70)	43.70 (12.09)	43.33 (12.08)	42.31 (13.70)
Mean eGFR (SD), mL/min/1.73 m^2^	42.35 (9.23)	43.03 (9.52)	42.26 (9.36)	42.54 (8.95)	43.03 (9.52)
Bias, ml/min/1.73 m^2^ ^a^	−1.24	0.71	−1.52	−0.94	0.71
IQR, ml/min/1.73 m^2^ ^b^	12.53	9.99	13.53	10.68	9.99
P_30_, %^c^	79.61	77.27	78.99	81.82	77.27
RMSE	9.69	8.57	9.86	9.27	8.57

Abbreviations: GFR, glomerular filtration rate; mGFR, measured GFR; eGFR, estimated GFR; IQR, interquartile range; RMSE = root mean square error.

^a^ Bias refers to measured GFR minus estimated GFR.

^b^ IQR refers to the 25–75th percentile.

^c^ P30 refers to percentage of GFR estimates that are within 30% of measured GFR.

External-validation of Xiangya-s Equation was performed in SXH and FXH centers. In both centers, Xiangya-s equation generally satisfied the requirements for guidelines (P_30_, 78.45% for SXH center and 79.05% for FXH center, respectively, Table 3). Notably, the external validation results of inpatients from FXH center (Supplementary Table S6) suggested that Xiangya-s equation yielded the greatest P_30_ of 80.42% in Uighur subgroup, indicating that the Xiangya-s equation had little racial differences in China.

**TABLE 3:** Performance of eGFR equations in the inpatients of external validation sample.

Equation	SXH center (mGFR = 43.81 ml/min/1.73 m^2^)					FXH center (mGFR = 42.80 ml/min/1.73 m^2^)				
	eGFR, ml/min/1.73m^2^	Bias^a^, ml/min/1.73m^2^	IQR^b^, ml/min/1.73m^2^	P_30_ ^c^, %	RMSE	eGFR, ml/min/1.73m^2^	Bias^a^, ml/min/1.73m^2^	IQR^b^, ml/min/1.73m^2^	P_30_ ^c^, %	RMSE
CG	46.94	3.14	20.07	57.46	18.66	68.11	20.49	31.54	39.79	30.52
MDRD	48.83	5.02	20.63	58.01	18.35	66.44	20.24	28.43	41.24	28.77
a-MDRD	51.89	8.09	22.50	54.70	19.47	70.69	24.48	27.10	35.84	30.47
c-MDRD	63.99	20.18	28.02	40.33	24.16	87.16	40.95	21.97	20.82	37.19
MDRD_(CN)_	54.54	10.73	25.08	49.72	21.92	75.88	29.68	28.39	29.64	34.53
CKD-EPI_cr_	51.56	7.75	24.15	53.59	19.52	67.61	21.41	24.38	36.63	26.38
CKD-EPI_(CN)_	56.72	12.91	27.05	46.96	21.50	74.38	28.17	22.11	28.99	28.82
Asian modified CKD-EPI	61.73	17.92	33.28	37.57	23.63	78.32	32.11	16.58	23.32	28.47
New modified CKD-EPI	60.77	16.97	29.33	38.12	19.94	72.65	26.44	10.34	24.37	21.86
Chinese MDRD 6	53.29	9.49	24.23	51.93	21.58	74.11	27.91	29.57	31.49	34.12
New modified MDRD	49.91	6.10	15.73	68.51	14.33	62.44	16.23	19.32	49.67	21.13
Ruijin	52.91	9.11	19.79	65.19	16.45	67.84	21.63	20.26	39.79	24.77
Xiangya-s	45.26	1.45	13.59	78.45	10.46	50.22	4.02	16.05	79.05	11.79

Abbreviations: CG, Cockcroft–Gault; MDRD, Modification of Diet in Renal Disease; CKD-EPI, Chronic Kidney Disease Epidemiology Collaboration; GFR, glomerular filtration rate; mGFR, measured GFR; eGFR, estimated GFR; IQR, interquartile range; RMSE, root mean square error; SXH, The Second Xiangya Hospital of Central South University; FXH, The First Affiliated Hospital of Xinjiang Medical University.

^a^ Bias refers to measured GFR minus estimated GFR.

^b^ IQR refers to the 25–75th percentile.

^c^ P30 refers to percentage of GFR estimates that are within 30% of measured GFR.

### Comparison of Equations Performance

Finally, 12 SCr-based equations, including CG (Cockcroft and Gault, 1976), MDRD (Levey et al., 1999), a-MDRD (Levey et al., 2000), c-MDRD (Ma et al., 2006), MDRD_(CN)_ (Ma et al., 2006), CKD-EPI_Cr_(Levey et al., 2009), CKD-EPI_(CN)_ (Teo et al., 2011), new modified CKD-EPI (Liu et al., 2014), Chinese MDRD 6 (Liao et al., 2011), Asian modified CKD-EPI(Wang et al., 2016), new modified MDRD (Pei et al., 2013), and Ruijin (Chang and Ye, 2015) equations, and 2 CysC-based equations, including CKD-EPI_CysC_ and CKD-EPI_Cr-CysC_ equations (Inker et al., 2012), were selected from 190 articles and evaluated in present CKD population (Li et al., 2019). As shown in the boxplot of mGFR and eGFR (Supplementary Figure S1), the closer median eGFR to mGFR and smaller IQR was found when comparing Xiangya-s equation to other formulas, indicating the superior model fitting effect.

In TXH center, Xiangya-s equation had the highest P_30_ of 79.91% for inpatients (Supplementary Table S7). Similar results could be seen in patients from other 2 centers (Table 3). For outpatients (Supplementary Table S8), Xiangya-s equation yielded the greatest accuracy (P_30_, 77.27%) among all eGFR equations in TXH, and its P_30_ was highest as compared to other 12 eGFR equations in FXH (70.19%). Importantly, when ranking these equations based on accuracy, the Xiangya-s equation was found to be the top 1 in all datasets of three centers.

Because equations we developed and validated was all based on SCr, we wondered whether other filtration markers, such as CysC, fitted this part of patients better than SCr. As shown in Supplementary Table S9, the performance was not improved as compared to the SCr-based equations. However, costs of CysC detection are approximately 12-fold higher than those for SCr in China.

To sum up, the Xiangya-s equation could estimate GFR more accurately when validated in the multi-center Chinese large CKD population, compared to reported SCr-based and CysC-based Asian eGFR equations. In general, for CKD patients who have undergone 99mTc-DTPA renal dynamic imaging, they should better monitor their mGFR levels continuously in subsequent life. Considering renal dynamic imaging is costly, cumbersome, and not so universal, they could estimate GFR more accurately by the Xiangya-s equation compared to other eGFR equations.

### DOACs Dosing Patterns and Associated Clinical Outcomes Using Renal Function Estimated by CG Equation

**FIGURE 2:**
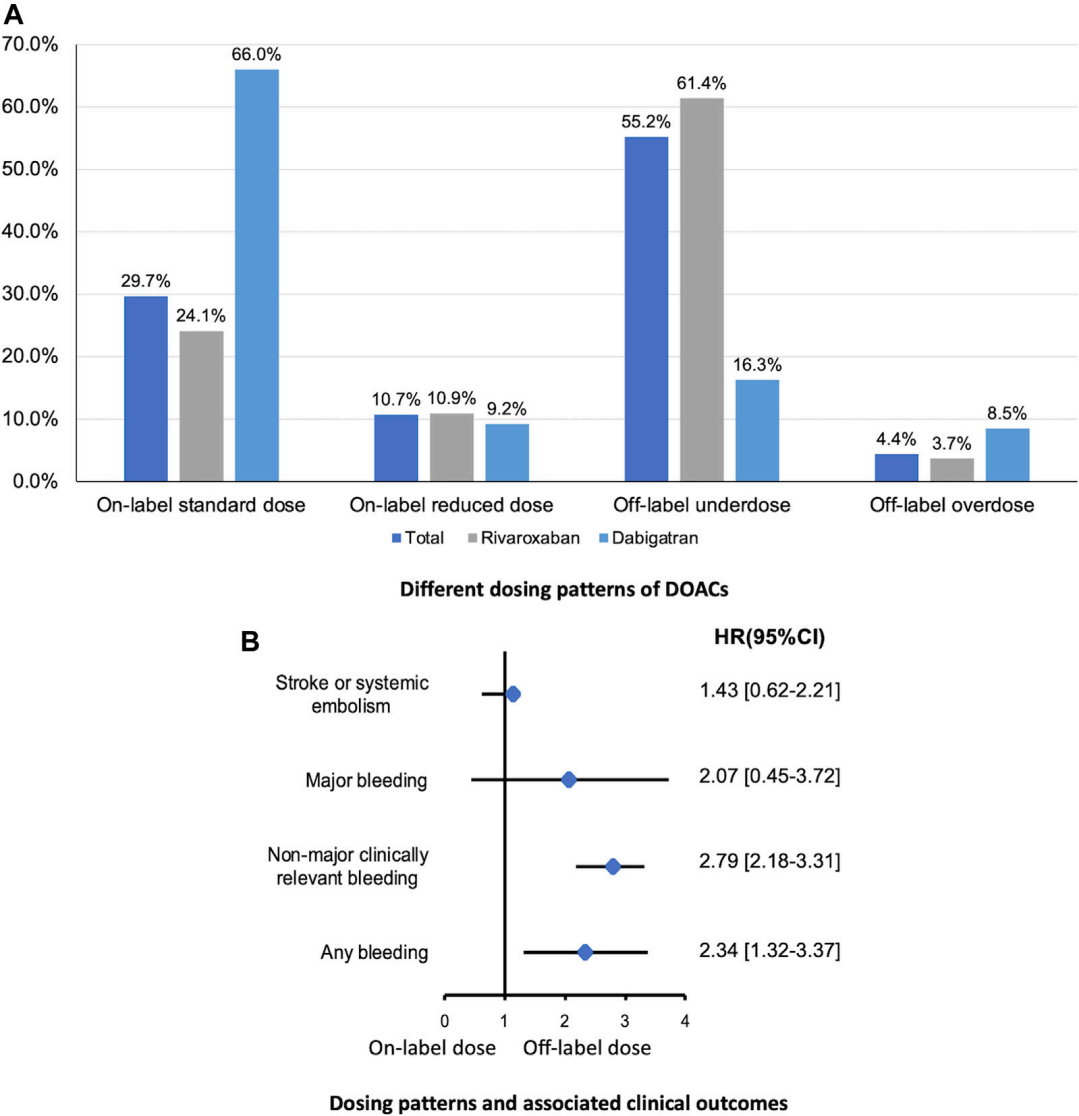
Different dosing patterns of DOACs **(A)** and associated clinical outcomes **(B)** in patients with eCrCl <50 ml/min calculated by CG equation. DOAC, direct oral anticoagulants; eCrCl, estimated creatinine clearance; CG, Cockcroft-Gault.

In present AF cohort, 24.1% and 56.1% of patients were prescribed an on-label standard dose of rivaroxaban and dabigatran, respectively. And, 72.3% and 25.6% of patients received a reduced dose of rivaroxaban and dabigatran, respectively. Thus, patients taking rivaroxaban were prescribed a reduced dose more frequently than those taking dabigatran. Among patients with a renal indication for dose reduction (*n* = 1,011), only 22.8% received on-label reduced doses. Among patients with no renal indication for dose reduction (*n* = 1,100), 44.1% received off-label reduced doses. Furthermore, 54 patients with the renal contraindication for DOACs still used rivaroxaban (*n* = 29) or dabigatran (*n* = 25) (Figure 2).

With respect to the clinical outcomes, no statistically significant difference was found in the risk of ischemic events comparing on-label dose vs. inappropriate dose, while rates of bleeding events were significantly higher in patients prescribed inappropriate dose of DOACs (Figure 2).

### Various Estimations for Renal Function Impact on Renal Function Categories and DOACs Drug Dosing

**FIGURE 3:**
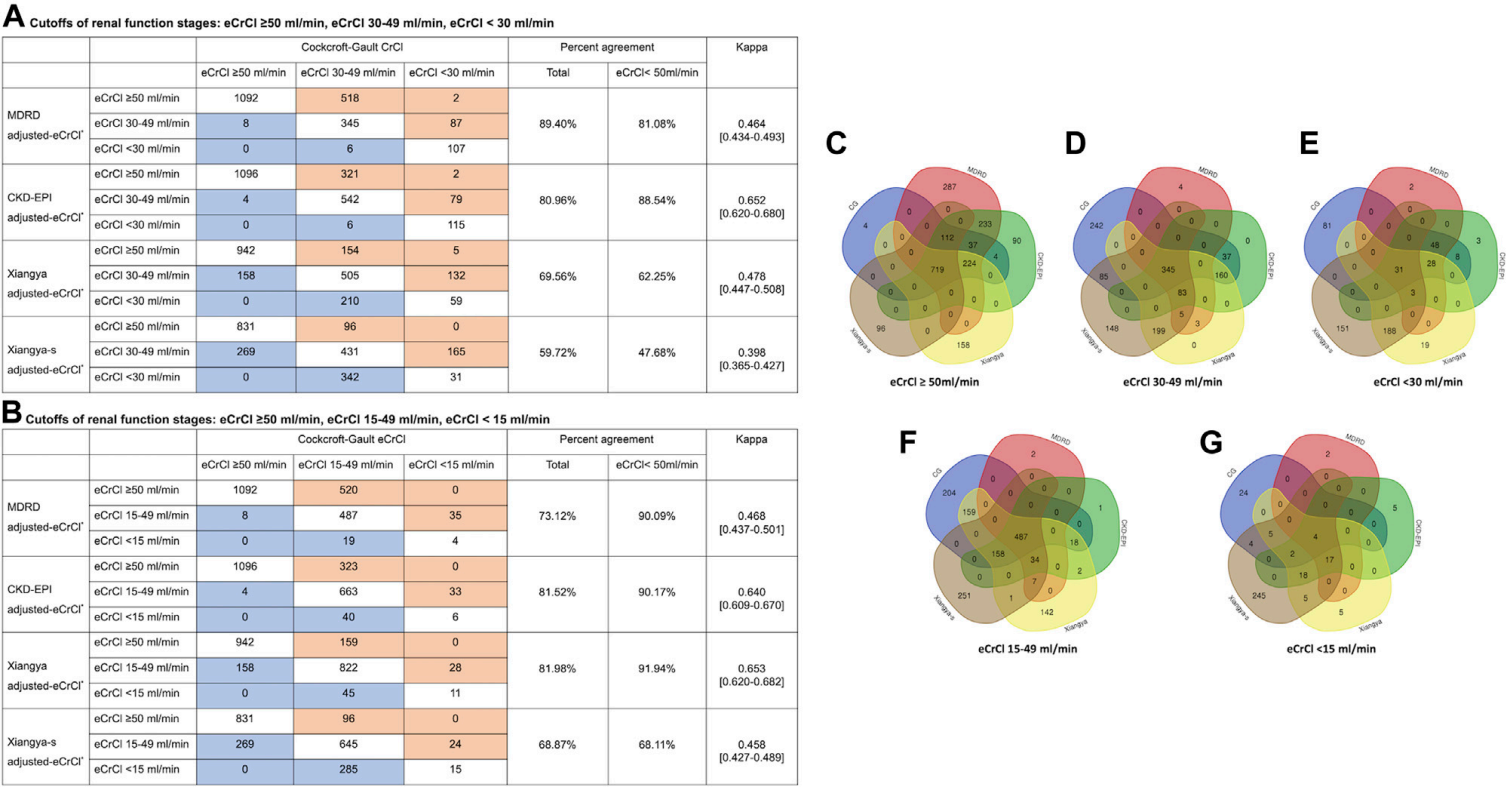
Impact of equations choices on patients’ renal stages **(A, B)** in confusion matrices and patient selection for eCrCl-based DOACs dosing adjustment **(C–G)**. The eCrCl cutoffs were as follows: **(A)** eCrCl ≥ 50 ml/min, eCrCl 30–49 ml/min, eCrCl < 30 ml/min; **(B)** eCrCl ≥ 50 ml/min, eCrCl 15–49 ml/min, eCrCl <15 ml/min; **(C)** eCrCl≥50 ml/min; **(D)** eCrCl 30–49 ml/min; **(E)** eCrCl <30 ml/min; **(F)** eCrCl 15–49 ml/min; **(G)** eCrCl <15 ml/min. DOACs, direct oral anticoagulants; eCrCl, estimated creatinine clearance; CG, Cockcroft-Gault; MDRD, Modification of Diet in Renal Disease; CKD-EPI, Chronic Kidney Disease Epidemiology Collaboration.

We compared the performance of Xiangya-s with Xiangya, and three most frequently used equations, CG, MDRD and CKD-EPI equations, in AF patients. The mean within-participant differences relative to CG equation were shown in Supplementary Figure S2. As shown in Figure 3, among patients with CrCl<50 ml/min estimated by CG equation, CrCl calculated by MDRD, CKD-EPI, Xiangya, and Xiangya-s equations classified 81.08%, 88.54%, 62.25% and 47.68% patients into the same renal function stage compared with CG equations, respectively. When the cutoffs of the different renal function stages were defined as: CrCl > 50 ml/min, CrCl 15–49 ml/min, and CrCl < 15 ml/min, similar results were found (Figure 3).

Furthermore, as shown in Figure 3, the five equations only agreed on drug-dose adjustment in 1.19%, 5.52%, 33.22%, 26.32%, and 36.61% of potentially impacted patients for eCrCl cutoffs of <15, <30, 15–49, 30–49, ≥50 ml/min, respectively.

### Clinical Outcomes Associated With Reclassification to Different Renal Function Stages

**FIGURE 4:**
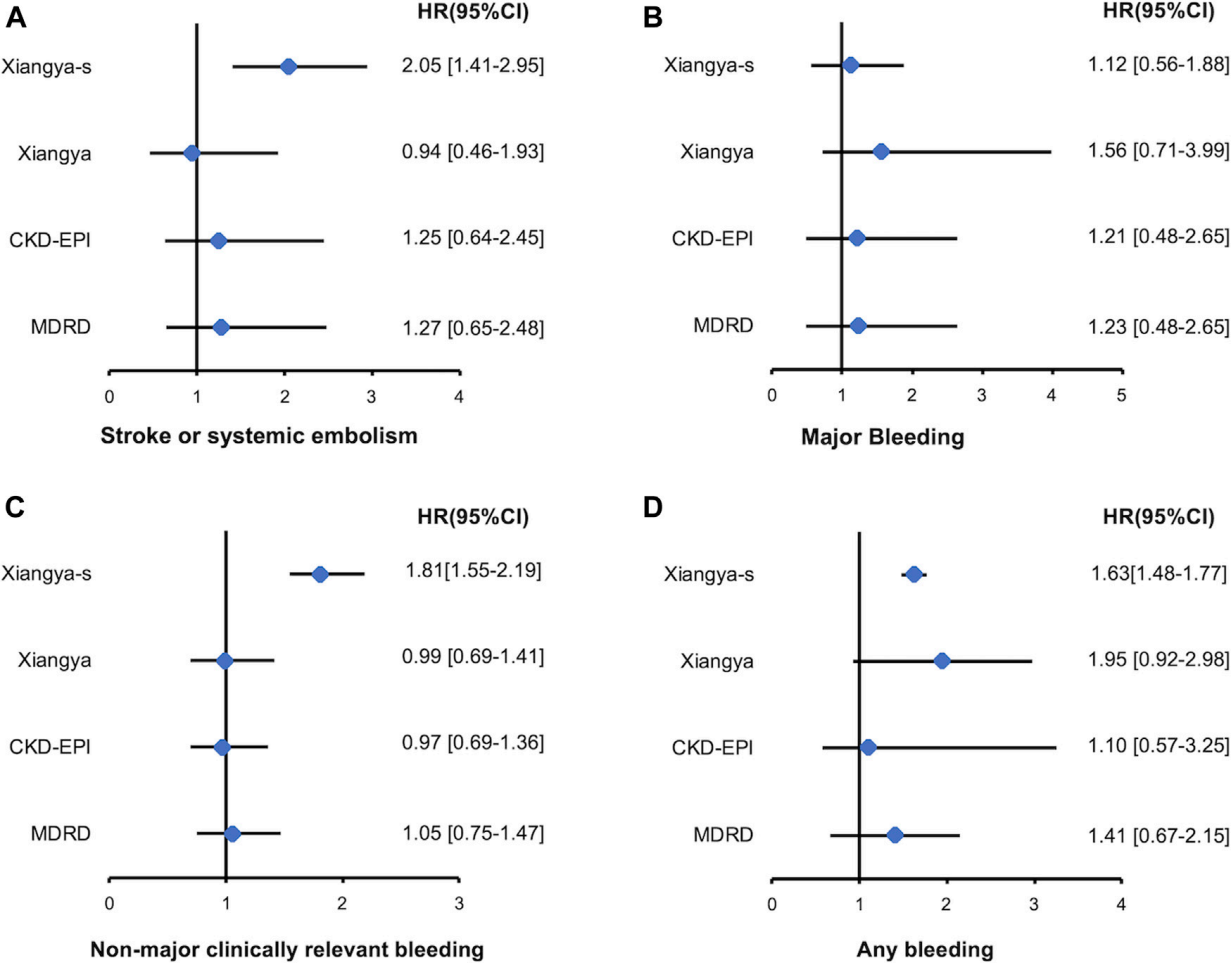
Stroke or systemic embolism **(A)**, major bleeding **(B)**, non-major clinically relevant bleeding **(C)**, and any bleeding **(D)** events associated with re-classified to a higher or lower renal function stages by MDRD, CKD-EPI, Xiangya and Xiangya-s equation compared with CG equation in patients with eCrCl <50 ml/min. eCrCl, estimated creatinine clearance; CG, Cockcroft-Gault; MDRD, Modification of Diet in Renal Disease; CKD-EPI, Chronic Kidney Disease Epidemiology Collaboration.

Among patients with eCrCl<50 ml/min, only reclassification to a higher or lower stage of renal function by Xiangya-s equation can better predict the S/SE events [2.05, 95%CI 1.41–2.95], non-major clinically relevant bleeding events [1.81, 95%CI 1.55–2.19] and any bleeding events [1.63, 95%CI 1.48–1.77] compared with CG equation, and no significant result was found for MDRD, CKD-EPI or Xiangya equations. Reclassification by MDRD, CKD-EPI, and Xiangya equation had no significant HR for all of the clinical outcomes (Figure 4). However, in patients with eCrCl ≥50 ml/min, reclassification to a different stage of renal function by Xiangya equation can appropriately predict S/SE events [1.18, 95%CI 1.10–1.24], non-major clinically relevant bleeding [1.71, 95%CI 1.14–2.28], and any bleeding [1.68, 95%CI 1.05–2.31] events compared with CG equation, and statistically significant fashion was found when predicting the non-major clinically relevant bleeding [2.04, 95%CI 1.18–3.53] and any bleeding [1.97, 95%CI 1.17–3.35] events for reclassification by MDRD (Supplementary Figure S3).

## Discussion

The principal finding of our study is that Xiangya-s equation performs better in estimating GFR of Asian CKD patients than commonly used equations and may assist in guiding dosage adjustment to reduce the associated adverse outcomes.

Inaccurate estimation of renal function may lead to inappropriate dosage adjustment, resulting in serious adverse events. However, in our previous study, none of the existing equations can precisely estimate the renal function in Asian patients with CKD (Li et al., 2019). Therefore, it seems to be crucial to develop a novel approach to assist in the dosing adjustment in patients with CKD. With those considerations as a backdrop, we firstly developed a novel equation in a multi-center with multi-ethnic Chinese population with mGFR<60 ml/min/1.73 m^2^. In this study, P_30_ for all of the existing eGFR equations was far below 75%. KDIGO 2012 CKD Guideline suggests measuring CysC and estimating GFR by the CysC-based CKD-EPI equations specifically when eGFR based on SCr is less accurate. Hence, we evaluated the CKD-EPI_CysC_ and CKD-EPI_Cr-CysC_ equations proposed by the guideline in those with mGFR <60 ml/min/1.73 m^2^. But performance did not get improved in comparison to the SCr-based equations. The external validation of Xiangya-s equation proved its excellent performance (P_30_, 79.61%) and consistence. Therefore, for those who had undergone renal dynamic imaging measurement with mGFR <60 ml/min/1.73 m^2^, we suggest calculating their following GFRs by Xiangya-s equation after performing a renal dynamic imaging test to guide clinical treatment. This could make them assess renal function much more accurately than other equations.

Off-label use of DOACs is a common phenomenon across the world. In the FANTASIIA Registry, the reduced dose of DOACs was prescribed to 44% of patients (57% for dabigatran, and 34% for rivaroxaban) in an adult population of Spanish patients with AF on anticoagulant treatment (Ortiz et al., 2017). In the CODE-AF registry, more than one-third of the study population was prescribed an off-label reduced dose of DOACs (Lee et al., 2019b). What’s more, compared to Western populations, the clinicians prefer to prescribe a reduced off-label dose of NOAC more frequently than in the western countries (Lee et al., 2019b). In a nationwide retrospective cohort study of consecutive patients with Nonvalvular Atrial Fibrillation (NVAF) taking DOACs using data collected from the Taiwan National Health Insurance Research Database, 87% and 90% patients were taking low-dose rivaroxaban and dabigatran, respectively (Chan et al., 2016b). In a cohort of East Asian patients, low-dose DOACs (75.1% for dabigatran, 59.7% for rivaroxaban, and 62.7% for apixaban) were more frequently used than standard-dose DOACs, resulting in lower clinical benefit (Cho et al., 2019). Several clinical factors may be associated with the high rate of underdose prescription in Asian patients. Asians are smaller in body size and body mass index compared with non-Asians. Therefore, low-dose DOACs may be potent enough at reducing thromboembolic events in Asians with low body mass (Chan et al., 2016b; Cho et al., 2019). Furthermore, the higher plasma concentration of dabigatran caused by advanced age and reduced creatinine was reported (Chan et al., 2016a). Similarly, pharmacokinetic profile of a 15-mg dose of rivaroxaban in Japanese patients was similar to that of a 20-mg dose in Caucasian patients (Hori et al., 2012). In present study, low doses were also more frequently used than the standard dose, especially for rivaroxaban (72.3%). However, the reasons for the high rate of underdose prescription in present cohort need further investigation.

Epidemiologic studies suggested that the prevalence of AF in CKD patients increased, and CKD has demonstrated to be an independent predictor of thromboembolism and bleeding events among AF patients (Steffel et al., 2018). Central to reduce the risk of adverse outcomes is appropriate treatment with anticoagulants. Given that each DOAC has a different threshold of renal function that mandates a reduction in dose (Steffel et al., 2018) and pivotal DOACs clinical trials only used CG formula to estimate renal function by calculating the eCrCl (Steffel et al., 2018), CG formula was recommended to assist in the dose adjustment of DOACs in CKD patients (Steffel et al., 2018). While the renal function estimated by CKD-EPI (Steffel et al., 2018) or MDRD (Levey et al., 2009) is more frequently used to make dosing decisions in routine clinical practice. The discordance in renal function stages and related different drug-dosing regimens classified by all of the equations mentioned have been reported. A recent study comparing several eGFR equations for dosing DOACs in AF patients reveals that eGFR obtained with the MDRD and the CKD-EPI equations was consistently higher than that obtained with the CG equation (Manzano-Fernández et al., 2015). And 50% discordance in dabigatran doses for MDRD or CKD-EPI equations in patients with GFR < 30 ml/min was shown, resulting in higher doses being given compared with the use of CG equation (Dowling et al., 2013). In present study, we also found that adjusted-eGFR calculated by MDRD and CKD-EPI was higher than that obtained with the CG equation. When applying the novel approach, Xiangya-s equation, to present AF cohort, reclassified to a higher or lower stage of renal function by Xiangya-s equation can predict the composite events of stroke or systemic embolism, non-major clinically relevant bleeding and any bleeding events compared with CG equation in patients with AF and CrCl < 50 ml/min. However, no significant result was found for MDRD, CKD-EPI, or Xiangya equation.

There also exist some limitations. First, our cohort is best described as Chinese adult CKD patients. We believe that our results will apply to this particular population, but comparison of equation performance is still needed to further validate. Second, the CysC-based equations were evaluated only in hospitalized patients from TXH center. This is because the cost of detecting CysC is high, causing the data of CysC in patients not easy to obtain. Third, different methods were used to get the actual renal function for developing different formulas to estimate kidney function. Thus, the system bias may not be avoided, contributing the differences in the performance of each equation on some extent. In addition, because Xiangya-s equation is based on creatinine level, it should be used with caution for patients who have abnormally high or low muscle mass. Fourth, only dabigatran and rivaroxaban were prescribed in our cohort. The performance of Xiangya-s equation to assist in other DOACs is still unknown.

In conclusion, our study demonstrates that none of the clinical commonly used equations can precisely estimate GFR in Asian patients with CKD, that may be associated with the risk of inappropriate dosing patterns related adverse outcomes. Xiangya-s equation can precisely estimate GFR in Chinese CKD patients and may be an alternative method of directly GFR measurement for patients who need consecutive monitoring of renal function, then letting them benefit from clinical treatment.

## Data Availability

The original contributions presented in the study are included in the article/Supplementary Material, further inquiries can be directed to the corresponding author.
